# Beneficial effects of GABA-producing potential probiotic *Limosilactobacillus fermentum* L18 of human origin on intestinal permeability and human gut microbiota

**DOI:** 10.1186/s12934-023-02264-2

**Published:** 2023-12-12

**Authors:** Sumanpreet Kaur, Preeti Sharma, Melinda J. Mayer, Saskia Neuert, Arjan Narbad, Sukhraj Kaur

**Affiliations:** 1 Department of Microbiology, Guru Nanak Dev University, Amritsar, India; 2 Gut Microbes and Health Institute Strategic Programme, Quadram Institute Bioscience, Norwich Research Park, Norwich, UK; 3 Present Address: Department of Medical Laboratory Sciences, Lovely Professional University, Jalandhar, India; 4 Present Address: East Genomics Laboratory Hub, Cambridge University Hospitals Genomic Laboratory, Hills Road, Cambridge, UK

**Keywords:** Gamma-aminobutyric acid, *Limosilactobacillus*, Psychobiotic, Metagenome, TEER

## Abstract

**Background:**

Gamma-aminobutyric acid (GABA) is a non-protein amino acid with neuroinhibitory, antidiabetic, and antihypertensive properties and is used as a drug for treating anxiety and depression. Some strains of lactobacilli are known to produce GABA and strengthen the gut barrier function which play an important role in ameliorating the effects caused by the pathogen on the gut barrier. The probiotic bacteria are also known to modulate the human fecal microbiota, however, the role of GABA-producing strains on the gut epithelium permeability and gut microbiota is not known.

**Results:**

In this study, we report the production of high levels of GABA by potential probiotic bacterium *Limosilactobacillus fermentum* L18 for the first time. The kinetics of the production of GABA by L18 showed that the maximum production of GABA in the culture supernatant (CS) occurred at 24 h, whereas in fermented milk it took 48 h of fermentation. The effect of L18 on the restoration of lipopolysaccharide (LPS)-disrupted intestinal cell membrane permeability in Caco-2 monolayers showed that it significantly restored the transepithelial electrical resistance (TEER) values, by significantly increasing the levels of junction proteins, occludin and E-cadherin in L18 and LPS-treated Caco-2 cells as compared to only LPS-treated cells. The effect of GABA-secreting L18 on the metataxonome of human stool samples from healthy individuals was investigated by a batch fermentor that mimics the conditions of the human colon. Although, no differences were observed in the α and β diversities of the L18-treated and untreated samples at 24 h, the relative abundances of bacterial families *Lactobacillaceae* and *Bifidobacteriaceae* increased in the L18-treated group, but both decreased in the untreated groups. On the other hand, the relative abundance of *Enterobacteriaceae* decreased in the L18 samples but it increased in the untreated samples.

**Conclusion:**

These results indicate that *Li. fermentum* L18 is a promising GABA-secreting strain that strengthens the gut epithelial barrier by increasing junction protein concentrations and positively modulating the gut microbiota. It has the potential to be used as a psychobiotic or for the production of functional foods for the management of anxiety-related illnesses.

**Supplementary Information:**

The online version contains supplementary material available at 10.1186/s12934-023-02264-2.

## Background

Human intestinal microbiota is considered an “organ” of the gastrointestinal tract that plays important roles in the host’s digestive process, epithelial cell development, and regulation of immunity, thereby contributing to the overall health of the host. An imbalance in the normal gut microbiota, known as dysbiosis, has been linked to number of inflammatory gut-associated diseases and metabolic illnesses [1]. Several studies have shown that the dysbiotic condition of the gut can be treated by using various therapeutic strategies such as intake of probiotics [2], prebiotics [3] and fecal transplantation of microbiota [4]. Probiotics are ‘live microorganisms that when administered in adequate amounts confer a health benefit on the host’ [5]. Probiotic bacteria belonging to the group lactic acid bacteria (LAB) such as *Limosilactobacillus reuteri* [6], *Lactiplantibacillus plantarum*, *Lactococcus lactis* and *Lactobacillus delbrueckii* [7] have been successfully used for the management and treatment of dysbiosis-related illnesses due to their ability to modulate the microbiota of the gut. Other benefits imparted by probiotic bacteria to the host include colonization resistance against pathogens and strengthening of the tight junction barrier of the gut epithelium [8]. Alteration of the diversity of the microbiome is associated with altered expression of junction proteins that further changes the permeability of the gut epithelial membrane [9]. Disruption of gut epithelial barrier is associated with the pathogenesis of lifestyle-related diseases such as diabetes [10], Crohn's disease [11, 12] and coeliac disease [13, 14]. Enteric pathogens disrupt the epithelial barrier either by causing alterations in the cytoskeleton or by the production of enzymes such as proteases that degrade tight junction proteins [15]. Whereas, probiotic bacteria are known to restore the gut epithelial barrier by inhibiting the growth of pathogens and by directly impacting the tight junction proteins of the epithelium.

Probiotic bacteria also benefit the host by secreting bioactive molecules. For example, some strains of lactobacilli have been shown to secrete essential amino acids [16], short-chain fatty acids [17], γ-aminobutyric acid [18] and exopolysaccharides [19] that play essential roles in the metabolism of a healthy host [20]. GABA is a non-protein amino acid that is produced in the human colon by gut microbiota due to the decarboxylation reaction carried out by the enzyme glutamate decarboxylase. GABA is one of the major inhibitory neurotransmitters in the mammalian central and peripheral nervous systems. Its oral intake has various beneficial effects including tranquilizing, hypotensive, diuretic and anti-diabetic [21]. Deficiency of GABA leads to neuropsychiatric disorders such as anxiety and depression. Therefore, extensive studies are being conducted to develop GABA-rich food supplements [22] that leverage manifold health benefits [23] such as neurostimulation [24], gut modulation [25] and cardioprotection [26]. Hagiwara et al. [27] demonstrated that GABA promotes the secretion of insulin from the pancreas, thereby preventing diabetes. GABA consumption can also regulate stress [28] and serum lipid levels [29]. Schuller et al. [30] demonstrated that GABA inhibits small airway-derived lung adenocarcinoma in mouse models. GABA production by *Lacti*.* plantarum* [31, 32]*, L*.* brevis* [33], *Lacticaseibacillus paracasei* [34], *Lentilactobacillus hilgardii* [35] and *Len*.* buchneri* [36] have been reported. Pharma-GABA approved by the Food and Drug Administration (FDA) as a food ingredient [37] is produced by fermentation process using *Len*.* hilgardii* K-3 [35]. The effect of GABA-producing lactobacilli strains on the gut microbiota and gut epithelium is not known.

In our previous study [38], the probiotic properties and safety aspects of lactobacilli strains isolated from the stool samples of healthy children was studied. The cell-free culture supernatant (CS) of L18 exhibited antibiofilm properties against *Vibrio cholerae* and *V. parahaemolyticus*. In this study, the GABA-producing potential of the isolated lactobacilli was compared. Further, the effects of *Li*.* fermentum* L18 on the permeability of colonic epithelial cell membrane and the modulatory effects of L18 supplementation on the metataxonome of the human fecal microbiome in batch fermentation was studied.

## Materials and methods

### Microorganisms and growth conditions

*Li*.* fermentum* L18 and other 7 lactobacilli were isolated and characterized (Additional file 1: Table S1) from the fecal samples of healthy children in the lab of Dr. Sukhraj Kaur in a previous study [38]. Lactobacilli were cultured in de Man Rogosa Sharpe (MRS; HiMedia Pvt. Laboratory, Mumbai, India) medium at 37 °C in anaerobic gas jars or in an anaerobic cabinet in an atmosphere of 5% CO_2_, 10% H_2_ and 85% N_2_ (Don Whitley, UK). The culture was preserved in 20% glycerol-containing MRS broth at − 80 °C and subcultured twice in MRS broth before using for the experiments. Log phase cultures were used for all the experiments.

### Cell lines and culture conditions

Human epithelial colorectal adenocarcinoma Caco-2 cell line was gifted by Dr. Sandra Tribola, Quadram Institute Biosciences (QIB), Norwich, United Kingdom. Caco-2 was cultured in Dulbecco’s Modified Eagle Medium (DMEM; Sigma-Aldrich Chemicals Pvt. Limited, St. Louis, USA) supplemented with 10% fetal bovine serum (Sigma-Aldrich) at 37 °C in 5% CO_2_-containing atmosphere. The media was changed regularly every 2 days. The Caco-2 cells with passage number 15 was used for the experiments.

Upon attaining > 80% confluency, the cells were harvested by trypsinization. For trypsinization, the adhered cells were washed twice with phosphate-buffered saline (PBS; pH 7) and 5 ml of 0.25% trypsin–EDTA solution (Sigma-Aldrich) was added to the cells and incubated at 37 °C for 5 min. The action of trypsin was stopped by the addition of 5 ml of medium. The trypsinized cells were collected in a sterile centrifuge tube and centrifuged at 200 g for 5 min at 37 °C. The pellet was suspended in 1 ml of medium and the cells were counted using trypan blue staining [39].

### Estimation of GABA

The production of GABA in CS by L18 was determined by an amino acid analyzer (Nexera X2, Shimadzu Asia Pacific Ltd, Japan). L18 was cultured in MRS broth containing 2% monosodium glutamate (MSG; HiMedia). To prepare the CS, lactobacilli were cultured for 16 h at 37 °C and the broth was centrifuged at 9000*g* for 10 min at 4 °C. The supernatant was then filter sterilized using syringe filters (0.2 μm) and stored at 4 °C until further use.

To study the production kinetics of GABA, CS was collected at different time points (24 h, 48 h and 72 h) and stored at 4 °C till further use. One microlitre of filter-sterilized CS was injected into the amino acid analyzer column, C18 (YMC-Triart, India), and detection was performed using fluorescent detector (RF-20A).

The production of GABA was also studied in 2% MSG-containing milk. The overnight grown culture of L18 was inoculated at 2% in 10% skim milk powder (HiMedia) containing 2% MSG and incubated for 48 h at 37 °C in anaerobic jars. After incubation, the fermented milk was treated with an equal volume of 24% trichloroacetic acid (HiMedia) and filtered through Whatmann 3 mm filter paper. The coagulated proteins were discarded and GABA levels in the filtrate were determined using the amino acid analyzer.

### Effect of lactobacilli on integrity of the epithelial cell barrier

The protective effect of L18 on the restoration of the integrity of LPS-disrupted confluency of colonic cell line Caco-2 cell monolayers was determined in vitro [40]. Caco-2 cells were seeded in the inserts of 24–transwell plates (Greiner Bio-One Ltd, United Kingdom) at the cell density of 0.33 × 10^5^ cells/cm^2^ in DMEM and incubated at 37 °C in 5% CO_2_-containing atmosphere. The media was changed on alternate days and the integrity of the monolayers was determined by measuring TEER of monolayers using a Millicell Electrical Resistance System (Millipore, Billerica, USA). The cell line was considered to be confluent after the TEER > 1000 Ω × cm^2^ was achieved. The cells were allowed to differentiate until day 14 and thereafter, the integrity of monolayers was disrupted by treating with 0.1 mg/ml LPS (Sigma-Aldrich). The integrity of the cell line in terms of TEER values was monitored after every 2 h. After 48 h of LPS treatment, L18 at concentration 10^7^ cells/ml was added to the apical wells and DMEM media without antibiotic was added to the basal wells. TEER values of the Caco-2 cells in different wells were measured up to 8 h.

### Effect of L18 on junction proteins of Caco-2 cells

The effect of L18 on the junction proteins of Caco-2 cells, occludin and E-cadherin was estimated by ELISA. After 4 h of the addition of L18 to the Caco-2 cells, trypsinization of the cells was performed and the collected cells were lysed by incubating the cells with chilled lysis buffer (0.2% triton-X prepared in 1X PBS, pH 7.2) for 5 min on ice followed by vortexing for 10 s. The lysed cells were centrifuged at 5000*g* for 20 min at 4 °C. The supernatant was collected and stored at − 20 °C till further use.

The levels of occludin protein and E-cadherin protein in the cell lysate of Caco-2 cells were determined using a Sandwich human occludin ELISA kit (Abbexa Ltd., Cambridge Science Park, Cambridge) and human E-cadherin ELISA kit (Invitrogen, Thermo Fisher Scientific Inc., USA), respectively as per the protocol of the manufacturer.

### Batch fermentation of fecal sample and analysis of microbiome composition

The modulatory effect of L18 on the human fecal microbiota was studied in a batch fermentation model [41, 42]. The in vitro batch fermentation was carried out for 24 h with continuous pH control 6.8 ± 2 of the set value in different vessels used to mimic the human colon. Fecal samples used in the colon model experiments were obtained from participants recruited in the QIB Colon Model study. Participants who were assessed to have normal bowel habits, regular defecation between three times a day and three times a week, with an average stool type of 3–5 on the Bristol Stool Chart, and no diagnosed chronic gastrointestinal health problems, such as irritable bowel syndrome, inflammatory bowel disease, or coeliac disease were enrolled onto the study. Demographic information was collected, and a brief health questionnaire was completed during the eligibility screening. Participants were asked additional questions immediately before donating a stool sample to confirm that they had not taken antibiotics or probiotics within the last four weeks, had not experienced a gastrointestinal complaint, such as vomiting or diarrhea, within the last 72 h, were not currently pregnant or breast-feeding, had not recently had an operation requiring general anesthetic, and were not taking iron or multivitamin supplements. The informed consent of all the participating subjects was obtained. The study was approved by the UK research ethics committee (REC15/LO/2169). Batch fermentation of feces was initiated by using prepared fecal inoculum that was processed by taking 10 g of fresh fecal samples from healthy volunteers and diluting 10 times in deoxygenated PBS (pH 7.7). The diluted feces were homogenized by using a Stomacher 400 (Seward, United Kingdom) operated at 230 rpm for 45 s. The overnight grown culture of *Li. fermentum* L18 in MRS broth was added to the vessel containing 135 ml of batch culture media (peptone water 2 g/l, yeast extract 2 g/l, NaCl 0.10 g/l, K_2_HPO_4_ 0.04 g/l, KH_2_PO_4_ 0.04 g/l, MgSO_4_.7H_2_O 0.01 g/l, CaCl_2_.2H_2_O 0.01 g/l, NaHCO_3_ 2 g/l, Tween 80 2 ml, glucose 10 g/l, vitamin K1 10 μl, cysteine HCl 0.5 g/l, bile salts 0.5 g/l, pH 7.0) at the final concentration of 2 × 10^6^ cells along with 15 ml of the processed fecal inoculum. At different time points (0, 8, 24 h), 13 ml samples were withdrawn and 1 ml of sample was used for colony-forming units (CFU) counting and remaining 12 ml sample were centrifuged at 3000*g* for 15 min and pellets were stored at − 20 °C. For CFU counting, samples were serially diluted in PBS inside the anaerobic cabinet. The total number of bacteria and the number of *Bacteroides, Bifidobacterium, Clostridium,* Lactobacilli, and *Enterobacter* spp. in each vessel were estimated by total CFU counts using Wilkins Chalgren, Bacteroides, Beerens, Clostridia, MRS, and McConkey selective agar plates, respectively (Oxoid, Basingstoke, Hampshire, UK). The samples were centrifuged at 3000*g* for 15 min and the pellets were stored at -20 °C for metataxonomic studies. Batch cultures were performed on three occasions.

### Metataxonomic profiling by 16S rRNA gene amplicon sequencing

To determine the bacterial composition of the fecal batch fermentation samples, paired-end 250 bp amplicon sequencing was carried out on an Illumina MiSeq instrument. The DNA from one of the fecal batch fermentation samples collected at different time points 0, 8, and 24 h was extracted by using FastDNA SPIN Kit (MP Biomedicals, California, USA).

The V4 region of the 16S rRNA gene was amplified with primers: 515F:5′-GTGCCAGCMGCCGCGGTAA-3′ and 806R: 5′-GGACTACHVGGGTWTCTAAT-3′ (Caporaso et al. 2012). The bar-coded 6-base ID tags were attached to both forward and reverse primers and were used to demultiplex samples. Sequence analysis was performed using the Quantitative Insights into Microbial Ecology (QIIME2 v.2018.8) pipeline [43]. Quality control and clustering of sequence variants were carried out using DADA2 [44]. Taxonomic assignment of the clusters was carried out using a naïve Bayes classifier pre-trained on the SILVA 132 rRNA database. Weighted UniFrac distances as well as observed operational taxonomic units (OTU) were determined to estimate beta and alpha diversity, respectively. Two-dimensional Principal coordinate analysis (PCoA) plots were generated with the Microsoft Excel plug-in XLSTAT using the weighted UniFrac distance matrix produced by QIIME2. Differential abundance testing between treatments/timepoints at the family level was performed using ANCOM [45].

### Statistical analysis

All the experiments were performed in triplicates and results shown are from three independent experiments represented as mean ± SD (standard deviation). The results were statistically analysed using unpaired Student’s *t*-test in GraphPad Prism 5.04 software.

## Results

### *GABA production by Li*.* fermentum L18*

**Fig. 1:**
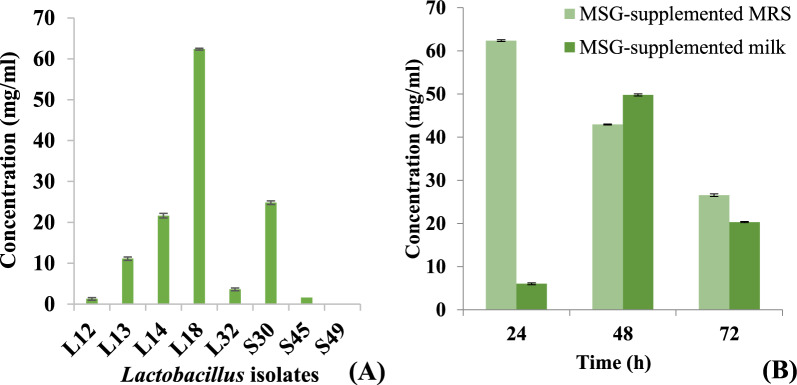
GABA production by **A** different *Lactobacillus* isolates in MSG (2%)-supplemented MRS (pH 5.5) and **B** *Li*.* fermentum* L18 MSG (2%)-supplemented MRS (pH 5.5) and in MSG-supplemented milk at different time points. The experiment was performed in triplicate. Bars are representative of the means and error bars represent the standard deviation

GABA-producing probiotic bacteria can serve as safe microbial source of GABA, that can be used to meet the increasing demands of GABA and GABA-enriched functional food. Lactobacilli isolated from healthy human stool samples were screened for the production of GABA in de Man Rogosa Sharpe (MRS) broth. Maximum production of GABA was observed in the CS fermented with for *Li*.* plantarum* L18 (Fig. 1A). The kinetics of GABA production by L18 was determined in MRS broth and milk samples. *Li*.* fermentum* L18 produced highest amounts of GABA (62.37 mg/ml) at 24 h, after which the concentration of GABA declined. At 72 h, the levels of GABA decreased to 24.45 mg/ml (Fig. 1B).

However, in L18-fermented milk supplemented with 2% monosodium glutamate (MSG), maximum GABA production (49.78 mg/ml) was obtained at 48 h whereas, at 24 h and 72 h, the amount of GABA detected was 6.012 mg/ml and 20.32 mg/ml, respectively. Thus, L18 has the potential of producing GABA-enriched fermented milk products (Fig. 1B).

### Effect of lactobacilli on the integrity of the epithelial cell barrier

**Fig. 2:**
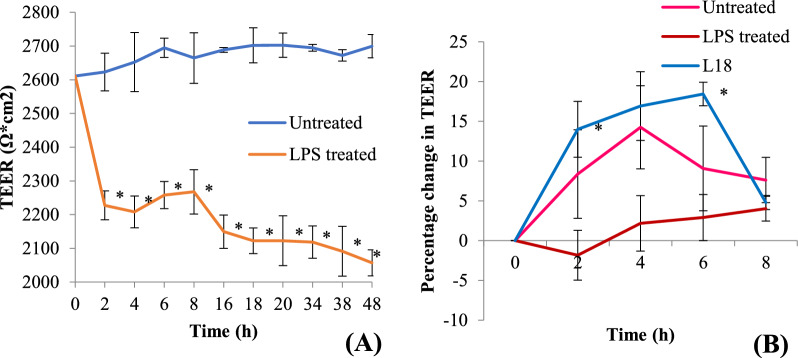
**A** TEER measurements of Caco-2 monolayers with and without treatment with LPS (0.1 mg/ml). **B** Percentage change in TEER values after addition of viable L18 cells to LPS-treated Caco-2 monolayers. The experiment was performed in triplicate. Significance of differences between untreated and LPS-treated was measured by using unpaired Student’s *t*-test. *Significance at *p* < 0.05

The effect of viable cells of L18 on the restoration of LPS-induced disruption of the human epithelial cell barrier was determined by using a human colonic cell line, Caco-2. The integrity and tightness of epithelial monolayers can be measured by using a well-established method, wherein, the TEER is determined across the confluent cell line. The addition of 0.1 mg/ml of LPS to Caco-2 monolayers cultured in the apical chamber of transwell plates resulted in a time-dependent decrease in the TEER values thereby implying the disruption of the integrity of the monolayer (Fig. 2A). Within 2 h of the addition of LPS, the TEER value was reduced by 14% that further decreased by 25% after 48 h. On the other hand, the TEER values of the untreated control monolayers remained almost constant over a period of 48 h (Fig. 2A).

After 48 h of the LPS treatment, viable L18 cells (2 × 10^6^ colony forming units (CFU)/well) were added to the Caco-2 monolayers (Fig. 2B). Within 2 h of the addition of L18 isolate, TEER value was significantly (*p* < 0.05) restored above that of the untreated control and it peaked at 6 h. After 6 h, the TEER values started decreasing, probably because of the multiplication of lactobacilli.

### Effect of lactobacilli on junction proteins

**Fig. 3:**
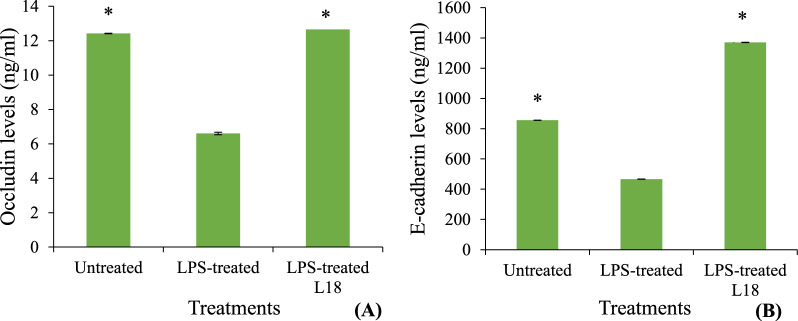
Concentrations of **A** occludin protein and **B** E-cadherin protein in Caco-2 cell lysates either untreated, treated with LPS (0.1 mg/ml) or treated with LPS and then incubated with viable cells (2 × 10^6^ CFU/well) of L18 isolate was determined by indirect Enzyme Linked Immunosorbent Assay (ELISA). The experiment was performed in triplicate. Bars are representative of the means and error bars represent standard deviation. Significant differences between the L18-treated sample and the only LPS-treated control were measured by using unpaired Student’s *t-*test. *Significance at *p* values < 0.05

In order to study the mechanism of restoration of the integrity of Caco-2 cell line by L18, the concentrations of tight junction protein, occludin and adherens junction protein, E-cadherin in Caco-2 cell lysates exposed to LPS in the presence and absence of L18 was determined. LPS treatment of Caco-2 cells led to a 50% decrease in the levels of occludin (Fig. 3A) and E-cadherin (Fig. 3B) as compared to the untreated controls at 52 h. The addition of viable L18 cells to the LPS-stimulated Caco-2 monolayers resulted in a significant (*p* < 0.05) increase in the concentrations of occludin (Fig. 3A) and E-cadherin (Fig. 3B) within 4 h of the addition of L18. occludin levels were restored similar to that of the untreated controls (Fig. 3A) whereas, the levels of E-cadherin increased to 1.6 times that of untreated control (Fig. 3B).

### Batch fermentation of fecal sample and analysis of microbiota composition

**Fig. 4:**
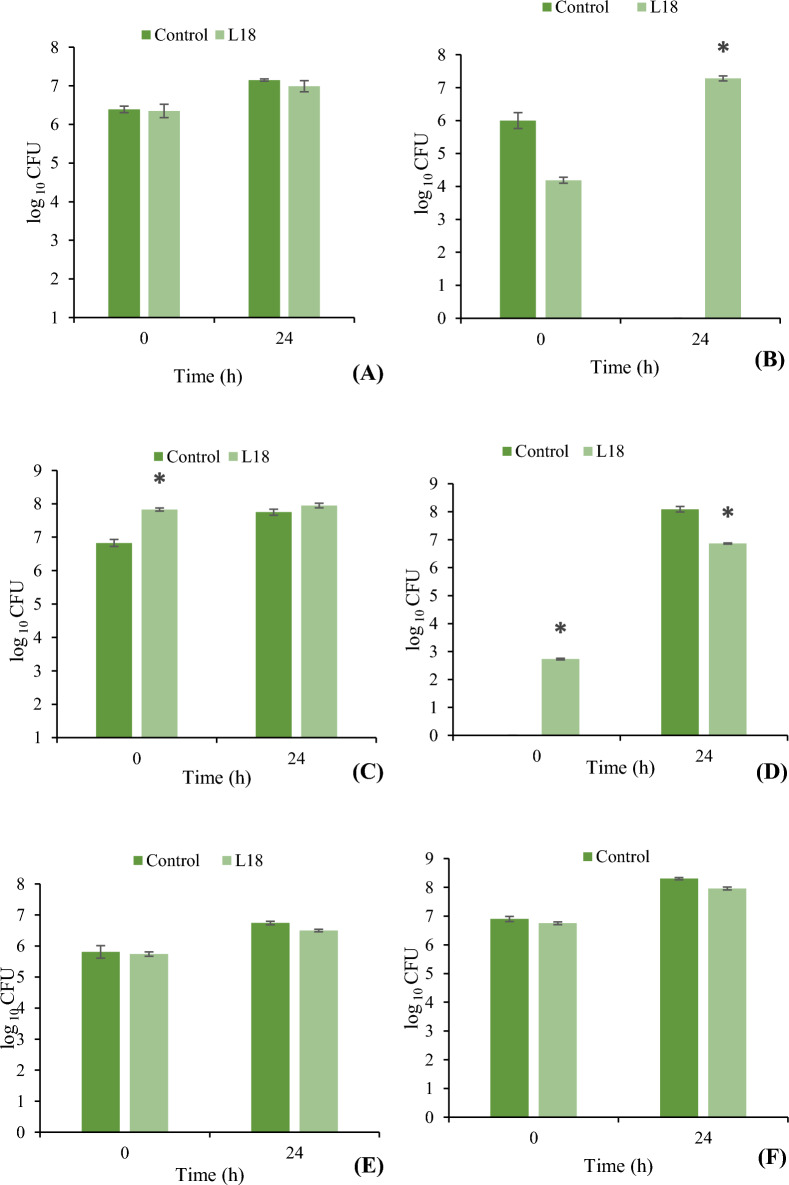
Culturable bacterial numbers in pH-controlled batch cultures of stool samples after 24 h of fermentation in the presence and absence of *Li*.* fermentum* L18. **A** *Bacteroides* **B** *Bifidobacteria* **C** Lactobacilli, **D** *Enterobacteriaceae* **E** *Clostridia* **F** Total anaerobes. Bars are representative of the means of three experiments and error bars represent standard deviation. *Significance (*p* < 0.05) was measured by using unpaired Student’s *t-*test

The effect of the addition of *Li*. *fermentum* L18 on the composition of the fecal microbiota in a batch fermenter was determined by both standard plate counting method as well as 16S rRNA gene sequencing of stool samples after 0, 8 and 24 h of fermentation. Colony-forming units (CFU) counts obtained on various selective agar media showed that the addition of L18 resulted in significant (p < 0.05) increase in *Bifidobacteria* at 24 h as compared to the control group (Fig. 4B). On the other hand, significant (p < 0.05) reduction in *Enterobacteriaceae* was observed in L18-supplemented group as compared to control group (Fig. 4D). No significant differences were observed in the CFU count of *Bacteroides* (Fig. 4A) Lactobacilli (Fig. 4C), *Clostridium* (Fig. 4E) and total anaerobes (Fig. 4F) in both the groups.

The metataxanome of the stool samples fermented in the presence or absence of L18 was evaluated at 0, 8 and 24 h and the data was compared. The sequence reads obtained per sample ranged from 193,274 to 309,057. After quality filtering, the sequence reads in range 159,453 to 250,226 were retained for further analysis. The operational taxonomic units (OTU) generated from the samples were in the range 345 to 557.

**Fig. 5:**
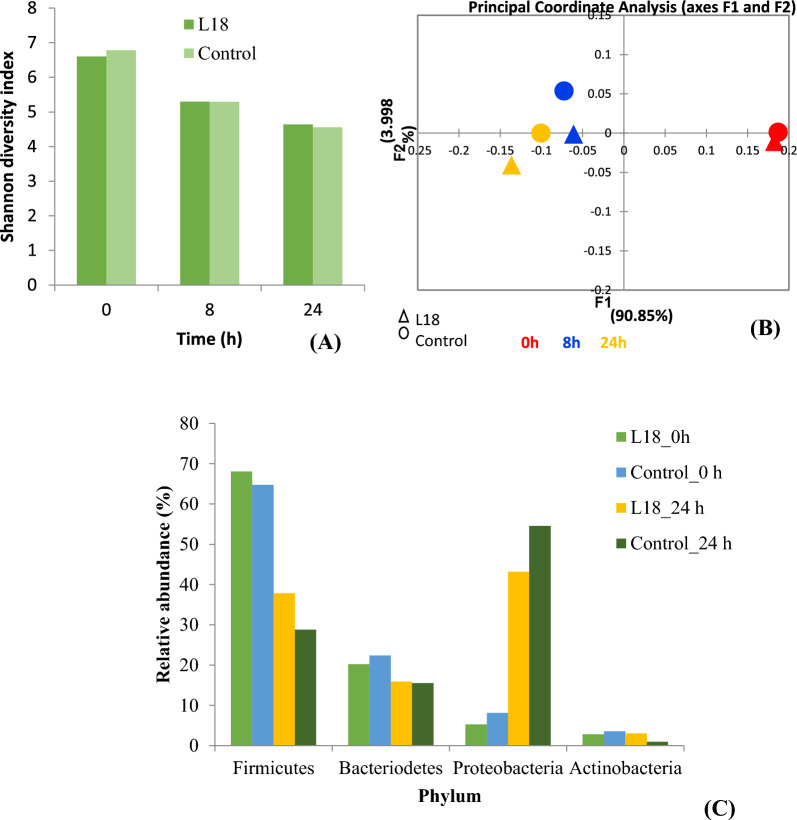
Showing changes in microbiota composition in batch fermentation. **A** Shannon diversity index. **B** Principal coordinate analysis (PCoA) score plot based on weighted UniFrac distances. **C** Relative abundances of the four predominant phyla in the fermentation samples at 0 h and 24 h

The alpha diversity of the samples at 0, 8 and 24 h was determined in terms of the Shannon index that takes into account species richness and evenness. The Shannon index decreased with time in both the groups but no differences were observed between the two groups (Fig. 5A).

Further, PCoA analysis was performed to compare the microbial diversity at the phylum or family level between the two groups and at different time points. A PCoA score plot based on weighted UniFrac distances showed separate clustering of 8 h samples from the 0 h samples along the F1 axis (90.85% variability), thereby indicating decrease in diversity over time in both the L18-treated and the control groups (Fig. 5B). Controls and L18-treated samples at both the time points 8 and 24 h separated primarily along the F2 axis, that explains only 3.99% of the variability between the samples, therefore indicating that the addition of L18 has a less strong effect on the community composition than time.

The relative abundance of 4 predominant bacterial phyla was determined in the samples at 0 and 24 h. In the 0 h samples, Firmicutes predominated the fecal microbiota of both groups followed by Bacteroidetes, Proteobacteria, and Actinobacteria (Fig. 5C). After 24 h of fermentation, the relative abundance of Firmicutes and Bacteroidetes decreased and the levels of Proteobacteria increased in both samples. The increase in the Proteobacteria was 37.9% and 46.4% in the L18-treated group and untreated samples, respectively. The abundance of Firmicutes decreased in both groups by 30.2% and 35.7% in the L18-treated and untreated groups, respectively.

**Fig. 6:**
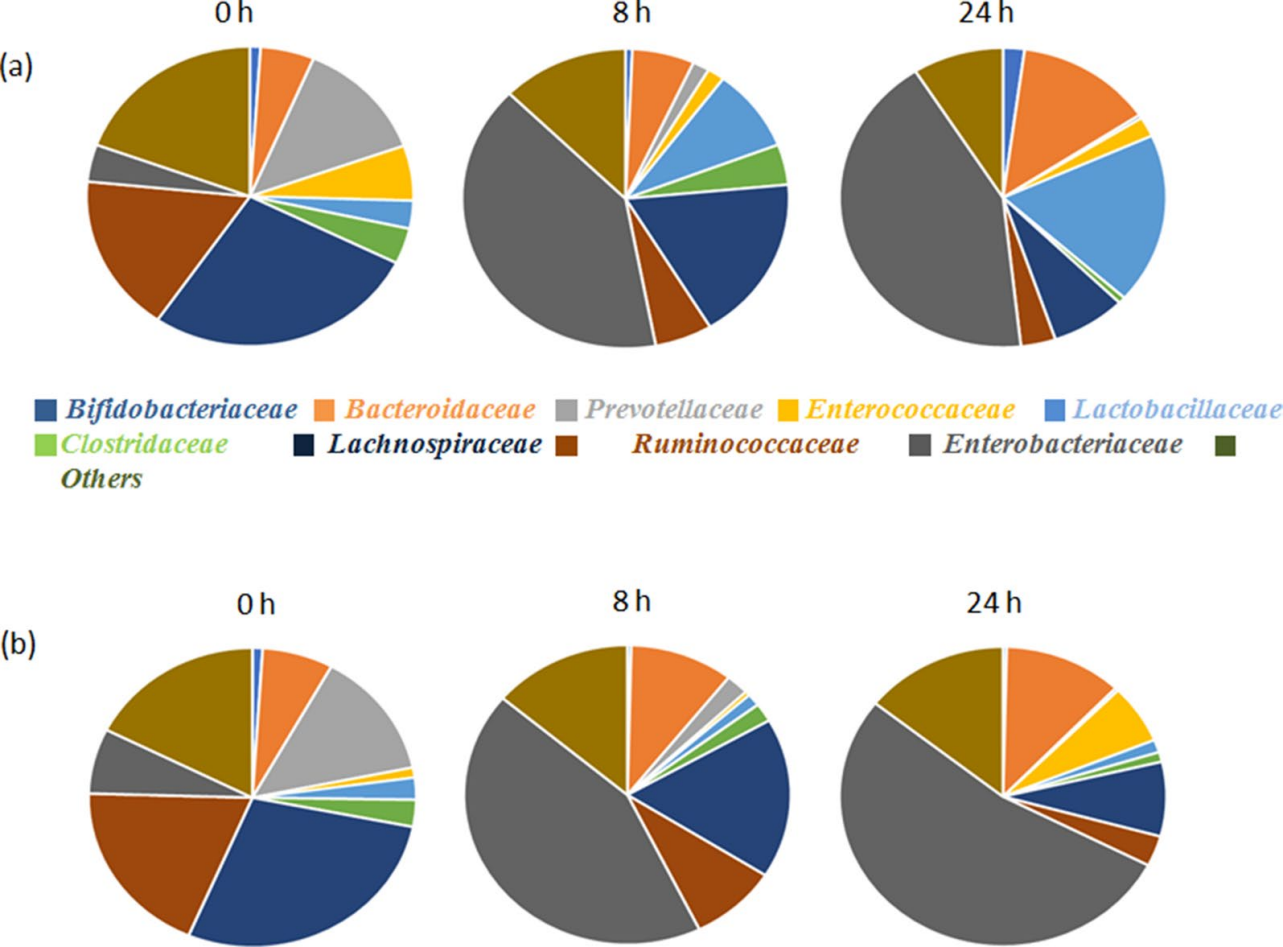
Relative abundance of different bacterial families in the samples obtained from fecal batch fermentations in the **a** L18-treated and **b** Untreated control group at 0, 8 and 24 h

The relative abundances of 9 different bacterial families in the L18-treated and untreated control were compared (Fig. 6). An increase in the relative abundance of *Lactobacillaceae* was observed over time in the L18-treated group (from 3% at 0 h to 18.4% at 24 h) as compared to the untreated group where a decrease in the abundance was observed (from 2.3% at 0 h to 1.3% at 24 h). Similarly, an increase in the relative abundance of *Bifidobacteriaceae* from 1% at 0 h to 2% at 24 h in the L18-treated group was observed; whereas, its abundance decreased from 0.9% at 0 h to 0.3% at 24 h in the untreated control. On the other hand, the relative abundance of *Enterococcaceae* decreased from 5.9% (0 h) to 2.14% (24 h) whereas in untreated control an increase from 1% to 6.3% was observed. The relative abundance of *Enterobacteriaceae* increased in both the groups at 24 h but percentage increase in untreated control (46%) was higher as compared to L18-treated group (38%). The relative abundance of *Clostridiaceae* decreased in both groups. However, the decrease was more in the L18-treated group (3.8% at 0 h to 0.7% 24 h) as compared to the untreated group (2.9% at 0 h to 1% at 24 h). No notable differences were observed in the relative abundance of the rest of the other genera.

## Discussion

Lactobacilli are one of the most frequently used probiotic groups due to their ‘Generally Regarded As Safe’ status. The health-promoting effects of lactobacilli is related to their ability to resist the colonization of pathogenic microbes due to the production of various beneficial metabolites such as organic acids, bacteriocins, etc. L18 is a potential probiotic bacterium that exhibited broad-spectrum, antimicrobial properties against both Gram-positive and Gram-negative bacteria owing to its ability to produce organic acids [38]. CS of L18 inhibited the biofilm formation of *V*.* cholera* and *V*.* parahaemolyticus* and caused the dispersion of preformed biofilms of *Vibrio* spp. The probiotic properties of L18 has already been reported in a previous study [38]. In this current study, we evaluated the ability of L18 to produce GABA and strengthen the intestinal epithelial barrier by enhancing the concentration of tight junction and adherens junction proteins.

The production of GABA by lactobacilli in medium supplemented with MSG has been reported earlier [46]. *L*.* brevis* is the most frequently reported species for GABA production. The highest production of 103.72 mg/ml of GABA in nutrient medium was reported from *L*.* brevis* NCL912 [47]. *L. plantarum* showed maximum levels of 0.20 mg/ml GABA in 3% MSG-supplemented MRS broth [32]. In another study, *Li. fermentum* produced 5.34 mg/ml of GABA in soya milk supplemented with 1.5% MSG. Thus, compared to these studies, L18 produced 10 times higher levels of GABA i.e., 62.37 mg/ml in MRS and 52 mg/ml in fermented milk. The levels of GABA decreased in CS by 2.3-fold after 72 h, which may be due to the action of the GABA-transaminase enzyme that reversibly converts GABA into succinic semialdehyde [48].

Further, the effect of L18 on the restoration of LPS-disrupted permeability of intestinal cell line was studied. The endotoxin LPS present in the cell wall of Gram-negative bacteria is known to increase epithelial permeability by disrupting the tight junction barrier [49]. Here we demonstrated that the addition of L18 to the LPS treated Caco-2 cell line resulted in improved tight junction barrier function after 2 h. However, after 6 h the reduction in TEER value was observed probably because of the overgrowth of bacteria. A study conducted by Fang et al. [50] showed that LPS treatment reduced the TEER of Caco-2 monolayers cocultured with peripheral blood mononuclear cells (PBMCs) by 9.9% after 48 h. The subsequent addition of *L. casei* led to a biphasic increase in TEER by 12.1% at 2 h followed by a decline at 6 h and the second peak of 86.7% increase was observed at 48 h. Another report by Ling et al. [51] demonstrated that the addition of *Bifidobacterium* cells along with LPS to Caco-2 monolayers significantly inhibited the reduction in TEER values as compared to LPS-treated monolayers. Further, the addition of *L. plantarum* DSM 2648 along with *E. coli* showed a 98.75% increase in TEER as compared to only *E. coli*-treated Caco-2 at 4 h followed by decline after 6 h [52]. Anderson et al. [53] showed that *L. plantarum* MB452 induced a 15–20% increase in TEER values of Caco-2 monolayers at 4 and 6 h as compared to untreated controls. Taken together these results indicate a valuable potential role for lactobacilli in protecting the gut and ameliorating epithelial cell damage.

Occludin is a 65 kDa integral plasma-membrane protein located in tight junctions that plays an important role in maintaining the barrier properties of tight junctions [54]. Whereas, E-cadherin is a calcium-dependent cell–cell adhesion glycoprotein that strengthens the cellular adhesion between the cells [55]. Therefore, we further studied the concentration of these proteins in LPS-treated Caco-2 cell lysates with or without the addition of L18 cells. LPS treatment of Caco-2 monolayers resulted in 1.9-fold and 1.8-fold reduction in the levels of occludin and E-cadherin proteins, respectively. The addition of L18 cells to LPS-treated Caco-2 monolayers increased the occludin and E-cadherin levels by 1.93-fold and 2.9-fold, respectively within 4 h. Thus, the observed increase in TEER values of Caco-2 monolayers upon addition of lactobacilli is possibly due to increased production of occludin and E-cadherin proteins that counteracted the LPS-mediated damage to the intestinal barrier. Similar results were demonstrated by another study, that showed that the addition of *L. plantarum* MB452 increased the mRNA levels of occludin by 1.36-fold [53]. Yang et al. [40] showed that the addition of *L. reuteri* cells to LPS-treated IPEC-J2 cells resulted in a twofold increase in mRNA levels of occludin. Karimi et al. [56] showed that the addition of *L*.* reuteri* cells to *E. coli*-treated IPEC-J2 cell monolayers caused 1.5- to twofold increase in E-cadherin levels. The mechanism through which L18 might enhance the levels of tight junction proteins could be related to its ability to secrete GABA. CS of GABA-secreting *L*.* brevis* was shown to increase the mRNA levels of tight junction proteins claudin and zona-occludin-1 and protect against IL-1beta-induced damage in a Caco-2 cell line [57]. In another study, purified GABA at concentration of 0.1–0.5 mM significantly increased the TEER levels indicating increased integrity of intestinal cell line Caco-2 [58]. GABA receptors are present on the intestinal cell lines and in response to binding GABA, the intestinal cells produce anti-inflammatory cytokines such as TGF-β and IL-10 and the production of proinflammatory cytokines is inhibited [59].

In vitro batch modeling of the digestive tract is used for evaluating the effects of potential probiotic and prebiotic substrates on fecal microbiota [60]. In this model, the microbiota from the stool samples of healthy humans is cultivated under simulated physiological conditions of the gut. Batch fermentation model to study the effect of *Li. fermentum* L18 on the composition of human fecal microbiome revealed that the addition of L18 had no effects on the alpha and beta diversities of the microbiome but differences in the relative abundance at the family level were observed. According to amplicon sequencing, after 24 h of fermentation, Proteobacteria dominated the microbiota followed by Firmicutes in both the samples; however, the increase in the L18-treated group was less as compared to untreated control. Proteobacteria is a phylum consisting of Gram-negative bacteria that includes members of *Enterobacteriaceae* family. Both CFU counts and 16 rRNA gene sequencing results have shown decrease in the counts of *Enterobacteriaceae*, which may account for less increase in Proteobacteria in lactobacilli-treated samples. The percentage of beneficial bacteria belonging to the families *Bifidobacteriaceae* and *Lactobacillaceae* increased by 1% and 18.4%, respectively in the L18-treated samples; whereas, they both decreased (by 0.6% and 0.94%, respectively) in the untreated sample. Increase in the *Bifidobacteria* was observed by both plating and 16S rRNA sequencing methods that indicate that L18 may be producing bifidogenic factors that promotes the growth of *Bifidobacteria*. In case of lactobacilli, although no differences in the counts between the groups was observed by the plating method but 16S rRNA sequencing showed that the relative abundance increased from 3% at 0 h to 18.45% at 24 h in L18 group. This may indicate that the added lactobacilli promoted the growth of unculturable species of lactobacilli which were unable to grow on MRS media. Similar to our results, Liu et al. [61] demonstrated that the addition of *L. acidophilus* (5 × 10^8^ CFU) to fecal microbiota in batch fermentation resulted in a significant increase by almost 1 log_10_ CFU in both *Bifidobacterium* and *Lactobacillus* after 24 h. Ogué-Bon et al. [62] studied the effect of *L. acidophilus* 14 150B on the canine fecal microbiota by FISH analysis. The addition of *L. acidophilus* (10^9^ cells) significantly increased the numbers of *Bifidobacterium* and *Lactobacillus* after 10 h of fermentation but after 24 h the number of *Bifidobacterium* decreased, whereas *Lactobacillus* numbers were higher as compared to the control. Further contrary to our study, the numbers of both *Clostridia* and *Escherichia coli* numbers increased after 24 h in the *L. acidophilus* group.

These results indicate that *Li*.* fermentum* L18 has several beneficial properties such as the ability to secrete high concentrations of GABA and to strengthen the intestinal gut barrier. It has the potential to be used as a psychobiotic or used commercially to produce GABA and GABA-enriched functional foods. L18 supplementation also positively modulated the microbiota of stool samples by increasing the abundance of beneficial gut microbes such as *Lactobacillus* spp. and *Bifidobacterium* and reducing the numbers of *Enterococcus* and *Streptococcus* spp. Further studies are required to determine the in vivo effects of L18 on the permeability and protection of the intestinal epithelium and to evaluate its immunomodulatory properties that could contribute to overall health of the host.

## Conclusion

Our results showed that GABA producing *Li. fermentum* L18 has potential to strengthen the gut barrier by upregulating the production of junction proteins. L18 also modulates the fecal microbiota by increasing the abundance of beneficial micro-organisms. Therefore, L18 can be explored as psychobiotic or can be used to form GABA-enriched functional foods.

### Supplementary Information

**Additional file 1: Table S1.** The species name and GenBank accession numbers of fecal lactobacilli isolates used in the study.

## Data Availability

Not applicable.

## Abbreviations

GABA: γ-Aminobutyric acid
TEER: Transepithelial Electrical Resistance
LPS: Lipopolysaccharide
CS: Culture supernatant
MSG: Monosodium glutamate
DMEM: Dulbecco’s Modified Eagle Medium
PBS: Phosphate-buffered saline
CFU: Colony forming units
OTU: Operational Taxonomic Units
PCoA: Principal Coordinate Axis
