# Supplementary material for: Beneficial effects of GABA-producing potential probiotic Limosilactobacillus fermentum L18 of human origin on intestinal permeability and human gut microbiota
Source: Microb Cell Fact. 2023 Dec 12;22:256. doi: 10.1186/s12934-023-02264-2 (PMC10717626; doi:10.1186/s12934-023-02264-2)
Supplement: Supplementary file 1 — Additional file 1: Table S1. The species name and GenBank accession numbers of fecal lactobacilli isolates used in the study. [file 12934_2023_2264_MOESM1_ESM.docx]

**Table S1.** The species name and GenBank accession numbers of fecal lactobacilli isolates used in the study

| **Isolate** | **Genus species** | **Accession number** |
| --- | --- | --- |
| L12 | *Lactiplantibacillus plantarum* | MH916634 |
| L13 | *Limosilactobacillus fermentum* | MK033891 |
| L14 | *Lactiplantibacillus plantarum* | KY582835 |
| L18 | *Limosilactobacillus fermentum* | MK033872 |
| L32 | *Lacticaseibacillus paracasei* | MK033960 |
| S30 | *Limosilactobacillus fermentum* | MK243452 |
| S45 | *Lactiplantibacillus pentosus* | KY780505 |
| S49 | *Lacticaseibacillus casei* | MK033992 |
